# Decision tree-based prediction of 5-year weight trajectories after bariatric surgery in adolescents and young adults: a retrospective cohort study from France and Sweden

**DOI:** 10.1038/s41366-026-02134-y

**Published:** 2026-07-01

**Authors:** Iva Gueorguieva, Celine Lespagnol, Patrick Saux, Kajsa Jarvholm, Erik Stenberg, Estelle Aubry, Jessica Amsellem-Jager, Regis Coutant, Myriam Dabbas, Robert Caiazzo, Naziha Khen-Dunlop, Gianpaolo De Filippo, Ibrahim Dagher, Beatrice Dubern, Catherine Terraz, Gabriele Maggioni, Philippe Preux, Helene Verkindt, Naima Oukhouya, Torsten Olbers, François Pattou

**Affiliations:** 1 Pediatric Endocrine Unit, CHU Lille, Univ Lille, Lille, France; 2 Translational research in diabetes obesity and metabolic diseases, U1190 Univ Lille, Inserm Institut Pasteur Lille, CHU Lille, Univ Lille, Inserm Institut Pasteur Lille, CHU Lille, Lille, France; 3 CNRS, Inria, Centrale Lille, UMR 9189 – CRIStAL, Université de Lille, Lille, France; 4 Childhood Obesity Unit, Skane University Hospital, Malmö, Sweden; 5 Department of Psychology, Lund University, Lund, Sweden; 6 Department of Surgery, Faculty of Medicine and Health, Örebro University, Örebro, Sweden; 7 Department of Pediatric Surgery Unit, CHU Lille, Université de Lille, Lille, France; 8 Pediatric Endocrine Unit, CHU Angers, Université d’Angers,, Angers, France; 9 Nutrition Obesity Department, Necker- Enfants Malades University Hospital, Paris, France; 10 Department of general and endocrine surgery, CHU Lille, Lille, France; 11 Pediatric Surgery Department, AP-HP Centre, Necker- Enfants Malades University Hospital, Paris, France; 12 Assistance Publique – Hôpitaux de Paris, Hôpital Robert-Debré, Service d’Endocrinologie et Diabétologie, Paris, France; 13 French Research Group in Adolescent Medicine and Health (GRMSA), Paris, France; 14 Minimally Invasive Digestive Surgery, Antoine-Béclère Hospital, Clamart, France; 15 School of Medicine, Paris-Saclay University, Orsay, France; 16 Pediatric Nutrition and Gastroenterology Department, Assistance Publique – Hôpitaux de Paris, Hôpital Armand-Trousseau, Sorbonne Université,, Paris, France; 17 Department of Biomedical and Clinical Sciences, Department of Surgery, Linköping University, Norrköping, Sweden

**Keywords:** Endocrinology, Obesity

## Abstract

**Background:**

Metabolic-bariatric surgery is an efficient therapy in selected adolescents with severe obesity. However, predicting the postoperative weight loss is challenging. A machine learning calculator predicting 5-year weight loss trajectory has been developed in adults, based on seven preoperative features. The aim of the present study was to adapt and test it in adolescents.

**Methods:**

Retrospective cohort study in patients aged 12–20 years undergoing Roux-en-Y gastric bypass (RYGB), sleeve gastrectomy (SG), or adjustable gastric band (AGB) in France and Sweden between 2001 and 2022. Primary outcome was the accuracy of 5-year BMI prediction, expressed as the median absolute deviation (MAD) between predicted and observed BMI. The model developed in adults, was trained in a subset of 80% of randomly selected adolescents, and secondly tested in the remaining 20%.

**Results:**

We enrolled a total of 2255 patients (1705 female [75.6%], 12–20 years [median 19]). Five-year follow-up data were available for 59% of French and 38% of Swedish patients. The median (IQR) 5-year total weight loss was 30.2% (23.9–38.6) for RYGB, 23.4% (13.7–32.8) for SG, and 13.4% (0.0–30.1) for AGB. The adapted model predicted the observed 5-year BMI with a MAD of 3.7 kg/m² (95% CI [3.3–3.9]). The accuracy of the model was maximal for bypass (3.2 kg/m² [3.0–3.7]), good for SG (3.9 kg/m² [3.1–5.0]), and lower for AGB (7.3 kg/m² [5.5–8.4]), and accuracy decreased with time and in adolescents under 19 years. Age, height, weight, and type of intervention influenced 5-year weight loss. Type 2 diabetes influenced weight loss until 2 years after surgery, but not later.

**Conclusion:**

The model had an acceptable accuracy for adolescents to predict 5-year postoperative weight loss trajectory. Accuracy decreased over time and was influenced by type of intervention and age. This calculator is available online: https://bariatric-weight-trajectory-prediction.univ-lille.fr/.

## Introduction

The prevalence of severe obesity is rising rapidly among children and adolescents [1, 2], exposing them to the early onset of comorbidities as well as substantial psychological and social burdens [3]. These difficulties often persist into adulthood, as 70% of affected youth remain in situation of obesity by the end of adolescence [4]. Although multidisciplinary lifestyle interventions have shown limited effectiveness in producing significant weight loss [5, 6], they remain essential for empowering patients and preparing them for more intensive treatments, including emerging pharmacotherapies and bariatric surgery [7–10].

In carefully selected adolescents with severe obesity, bariatric surgery remains the most effective option for achieving sustained weight loss and improving comorbidities when conventional approaches have failed [7, 9, 11–15]. Despite high-quality training programs in experienced clinical teams, surgical approaches still carry the risk of acute and long-term complications, as well as variable outcomes [16]. Clear communication about the expected degree of weight loss is therefore crucial. Among adolescents, unrealistic expectations can lead to postoperative disappointment and may exacerbate mental health issues following bariatric surgery [17].

Recently, a tool applying machine learning for bariatric weight trajectory prediction has been developed for adults and has been internationally validated [18]. The algorithm, based on decision trees, is able to predict 5-year postoperative weight loss trajectories, using only seven preoperative features: weight, height, type of intervention, age at intervention, smoking history, type 2 diabetes (T2D) status, and diabetes duration [19].

The aims of the present study were [1] to train and validate this predictive model in youth, in order to adapt it from adults to adolescents, and [2] to determine whether patient characteristics and the choice of surgical procedure—adjustable gastric banding (AGB), vertical sleeve gastrectomy (VSG), or Roux-en-Y Gastric Bypass (RYGB)—affect the accuracy of weight-loss prediction in adolescents compared with adults.

## Methods

### Study design and participants

In this international retrospective observational study, we used data from two cohorts of adolescents living with obesity and submitted for the first time to bariatric surgery (RYGB, SG, and AGB), one from France gathering patients from 5 academic centers, and one from Sweden based on the national registry. Participants were aged 12–20 years, and were followed up to 5 years after surgery. If patients had a subsequent bariatric intervention within 5 years following the first one, data were censored at the time of reoperation. Missing postoperative data were assumed to result from loss to follow-up. Patients with missing postoperative values were retained in the analysis and censored at the corresponding time points. No missing data were observed for baseline covariates.

In France, data were collected in the University Hospitals of Lille, Angers, and Paris (Bicêtre, Robert-Debré, Necker and Armand-Trousseau Hospitals) for the period from March 2001 to December 2022. Data from all patients under 18 years came from electronic or paper medical records. In Lille, we also enrolled participants from the ABOS cohort (NCT01129297) aged between 18 and 20 years. In order to reduce the number of missing data, some patients were also contacted by telephone and e-mail, particularly for long-term follow-up. In Sweden, virtually all patients undergoing metabolic, bariatric surgery are registered in the nationwide research and quality registry, SOReg (the Scandinavian Obesity Surgery Registry) [20]. Validated anonymous data from the SOReg were included in the current study.

Empirical weight-loss trajectories stratified by surgical procedure were compared between the French and Swedish cohorts to assess their compatibility. As the overall shapes and variability patterns were comparable, the cohorts were assumed to be sufficiently similar to justify a joint analysis.

The study protocol was approved by the Ethics Review Committee (n°2023-070) of the University of Lille. Non-opposition forms were sent out to all French participants by post. In Sweden, all patients are informed of the registry and that research will be conducted based on information from the registry (“opt-out”). Given the anonymous extraction of data, no additional ethics approval in Sweden was necessary.

Data analyzed for each patient at baseline included the seven clinical items which have been shown to be the most relevant for predicting weight loss in adults: height, weight, and age at surgery, smoking status, diagnosis, and duration of T2D. Smoking status was defined as “smoking” or “nonsmoking” at surgery. We then analyzed preoperative weight, assessed immediately before surgery, and postoperative weight measured at 12, 24, 36, and 60 months after surgery. Total weight loss (TWL) in percent was calculated as:

$${\rm{TWL}}=({\rm{{visit}}\;{\rm{weight}}{{\mbox{-}}}{\rm{preoperative}}\;{\rm{weight}})/{\rm{preoperative}}\; weight}\times 100$$

### Study outcomes

The primary outcome was the accuracy of 5-year BMI prediction, expressed as the median absolute deviation (MAD), between predicted and observed BMI expressed in kg/m². Secondary outcomes were weight loss prediction accuracy at earlier postoperative visits (at months 12, 24), expressed by MAD and normalized MAD as a percentage of BMI.

### Adaptation of the adult prediction model to teenagers’ cohort

For the purpose of this study, the French and Swedish cohorts were first merged into a single dataset, which was further divided into two subsets as follows. Among each of the three intervention subgroups (AGB, SG, RYGB), 80% of patients were randomly selected to form the training subset, and the remaining 20% were assigned to the testing subset. This assignment criterion ensures that both the training and testing subsets reflect the repartition of interventions observed in the merged cohort.

Following the methodology developed for adults [19], the present study used decision trees, a class of machine learning non-linear algorithms that are able to learn customized predictions depending on multiple variables. First, we specialized the existing tree-based prediction model [19] to teenagers, based on the seven attributes that were previously identified by the LASSO (least absolute shrinkage and selection operator) in adults [21], namely type of intervention, preoperative weight, height, age at intervention, smoking status, diabetes status and duration. This choice was motivated by two main considerations: these variables showed strong predictive performance in adults while being simple and routinely collected in clinical practice, and the adult dataset used for variable selection already included young adults aged 18–20 years, supporting their relevance for a younger population. We subsequently applied the CART algorithm on the training subset to learn adolescent-specific subgroups and predicted TWL. Regression trees were fitted using the CART algorithm implemented in the rpart R package. Model complexity was controlled by the cost–complexity pruning parameter (cp). For each analysis, a maximal tree was grown on the training data and subsequently pruned. The optimal value of cp was selected by minimizing the cross-validated relative error using the default 10-fold cross-validation procedure in rpart, and the corresponding subtree was retained. Finally, we compared the predicted weight loss at each time point after surgery and the actual values measured in patients from the testing subset.

### Statistics

The baseline variables used to predict the weight loss trajectories were extracted from the retrospective French and Swedish patients’ medical records. The prediction model consisted of decision trees, trained at each outcome date to predict percent of TWL from the baseline variables, using the classification and regression trees (CART) algorithm [22]. Predicted TWL were then converted into predicted weight and BMI. The accuracy of the predicted outcomes with respect to the true outcomes was reported in terms of MAD with 95% confidence intervals (CI) estimated by bootstrap (BCa method, *n* = 10,000 replications). The predictive model for adolescents was then compared with the adult predictive model [19], based on MAD. We also used Bland-Altman plots to evaluate model calibration and 1-sample t-tests to assess the presence of systematic bias.

Weight and BMI median (Interquartile Range [IQR]) 5-year trajectories of participants submitted to each operation were illustrated as a function of time using a nonlinear smoothing. Subgroup analyses were performed by splitting the study cohort by intervention, pregnancy status, age (younger vs. older than 19 years old) and AGB removal.

The analysis was performed using the R software version 4.3.1, the library rpart for the CART implementation. We used the Transparent Reporting of a Multivariable Prediction Model for Individual Prognosis or Diagnosis (TRIPOD AI) guidelines to report the prediction model’s development and validation.

## Results

The overall study enrolled a total of 2255 participants from France (*n* = 329) and Sweden (*n* = 1926).

**Fig. 1:**
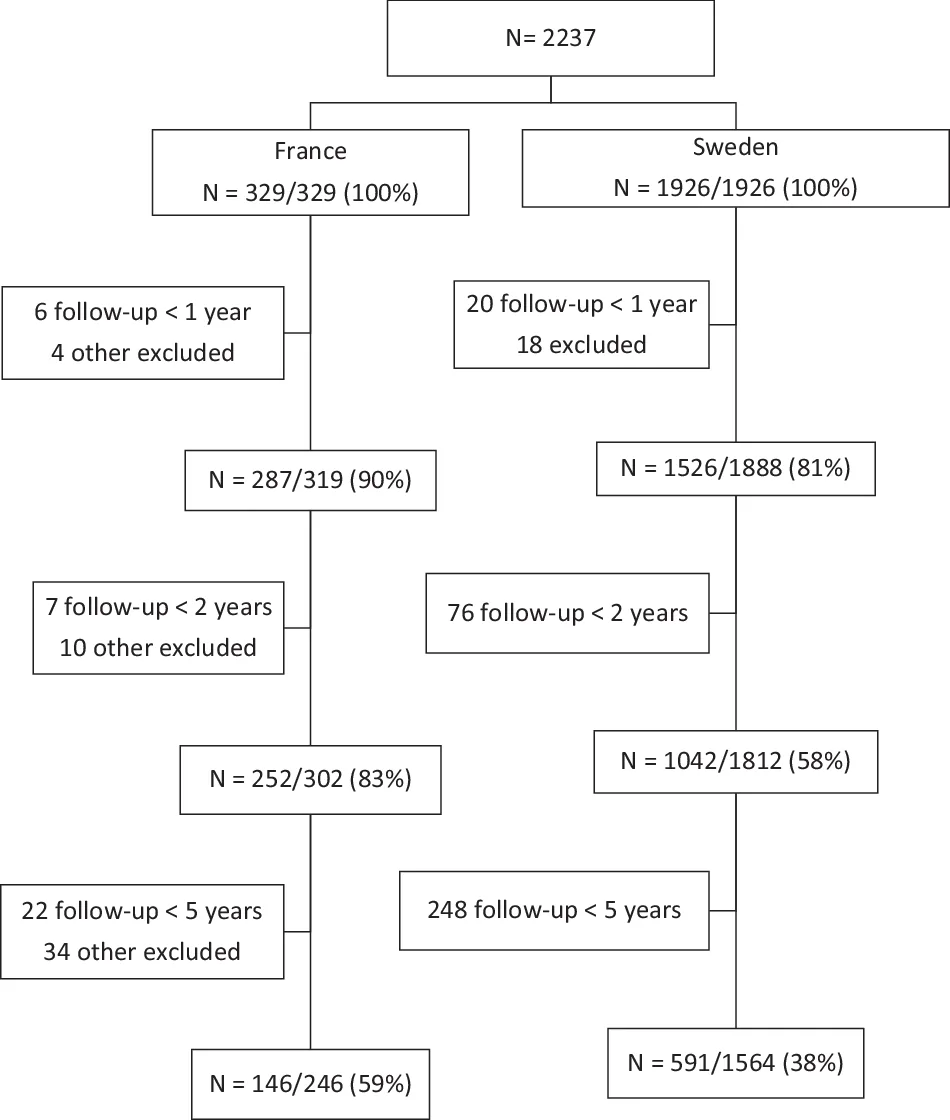
Flow chart for French (left boxes) and Swedish (right boxes) cohorts. Boxes on each line represent the number of patients with available data at each time point (baseline, 12, 24, and 60 months). Intermediate boxes represent the number of patients excluded at each time of assessment (see “Methods”).

Dropout rates and patients excluded for analysis are presented in a flow chart according to each follow-up time (Fig. 1). At 5 years, follow-up data were available for 59% and 38% of expected visits in French and Swedish cohorts respectively. In the French cohort, main reasons for excluding patients were: reoperation by another bariatric surgical technique; AGB removal; pregnancy within the 5-year period, and death. For the Swedish cohort, missing data at postoperative time points were assumed to be from patients lost to follow-up. The baseline characteristics of participants of each cohort and the proportion of each type of operation performed are shown in Table 1.

**Table 1:** Baseline characteristics (M0).

Cohort	France	Sweden	Merged
Sample size, *n*	329	1926	2255
Weight, kg, mean (SD)	128.4 (25.1)	127.5 (2.2)	127.3 (22.3)
Height, cm, mean (SD)	166.4 (12.4)	170.3 (8.8)	NA
BMI, kg m^−2^,mean (SD)	45.9 (7.8)	43.8 (5.7)	44.0 (6.0)
Sex = F, *n* (%)	252 (76.6)	1453 (75.4)	1705 (75.6)
Age, years, median (IQR)	17.4 (1.8)	19.0 (2.0)	19.0 (2.0)
Intervention			
AGB	163 (49.5)	5 (0.3)	168 (7.4)
SG	97 (29.5)	242 (12.6)	339 (15.0)
RYGB	69 (21.0)	1661 (86.2)	1730 (76.7)
others	0	18 (0.9)	18 (0.9)
T2D, *n* (%)	6 (2)	69 (3.6)	75 (3.4)
Smoker, *n* (%)	42 (13.0)	302 (15.7)	344 (15.2)

*AGB* adjustable gastric band, *BMI* body mass index, *F* female, *IQR* interquartile range, *NA* not available, *others* another surgical technique, *RYGB* Roux-en-y gastric bypass, *SG* sleeve gastrectomy, *T2D* type 2 diabetes.

During the study period, AGB was the most frequent intervention in France (50%), while in Sweden, the majority of patients underwent RYGB (87%). Eighteen Swedish patients (0.9%) had another surgical technique and were excluded. At baseline, individual weights and BMIs ranged from 72 to 235 kg and from 25.6 to 79.5 kg m^−^², respectively. The median age was 19 years, which corresponds to the WHO definition of the end of adolescence (10–19 years) [23]. Thus, the cohort included 1514 adolescents and, additionally, 741 young adults aged 19–20 years.

**Fig. 2:**
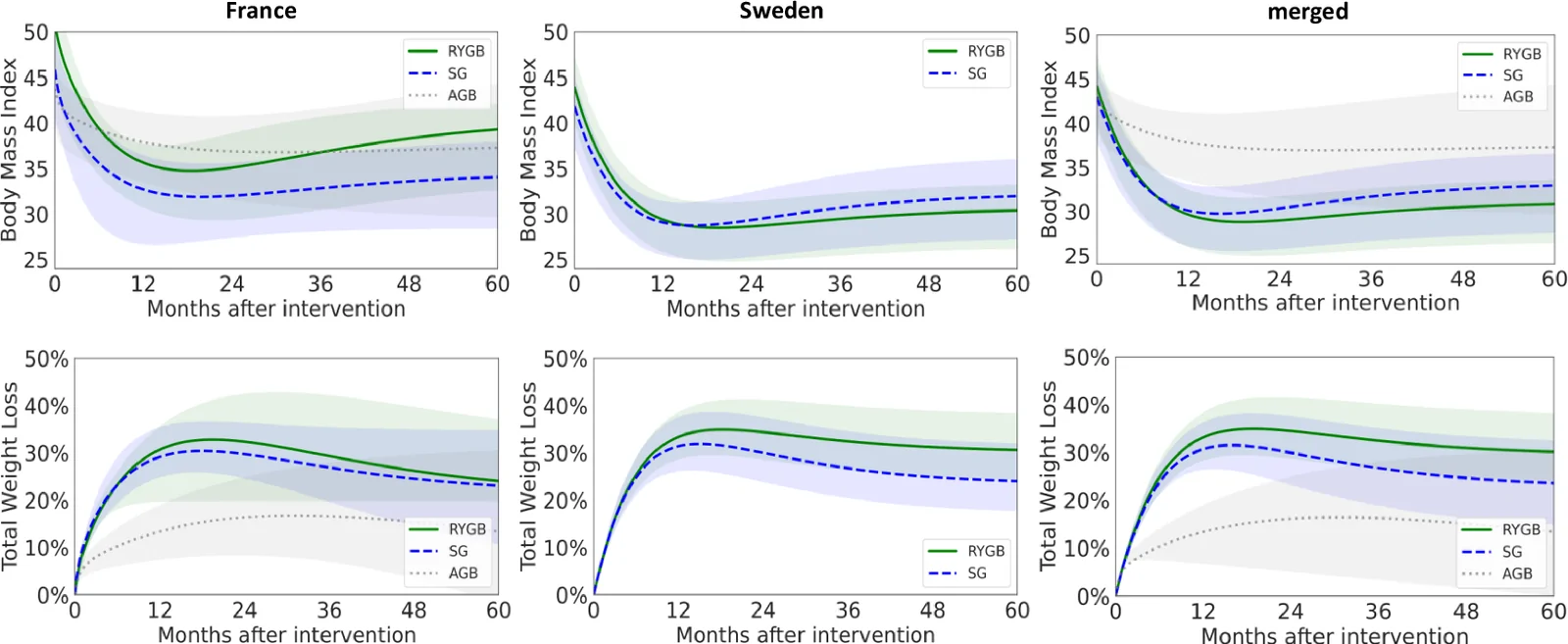
Body mass index (kg/m^2^) and total weight loss (% of preoperative weight) at 12, 24, and 60 months after surgery, according to each procedure in individual and merged cohorts. AGB adjustable bastric band, RYGB Roux-en-y gastric bypass, SG sleeve gastrectomy.

The overall trajectories of the median (IQR) BMI and TWL observed during the 5 years after each operation are shown in Fig. 2. Those data are shown first, for each country, and second, for the merged cohort.

At 5 years, the median TWL was 30.2% (IQR 23.9–38.6) for RYGB, 23.4% (IQR 13.7–32.8) for the SG and 13.4% (IQR 0.0–30.1) for the AGB. Although Swedish patients were overall older and with a lower BMI, the general shapes of weight loss trajectories for each operation were similar among cohorts. The nadir weight loss was reached between 1 and 2 years, followed by limited weight regain: median 14.5% (IQR 0.0–31.0) of maximal weight loss across operations. Weight regain was significantly (*p* < 0.001) greater after sleeve gastrectomy 27.1% (IQR 0.0–49.3), compared to gastric bypass 13.9% (IQR 0.0–27.3) (Fig. 2).

**Fig. 3:**
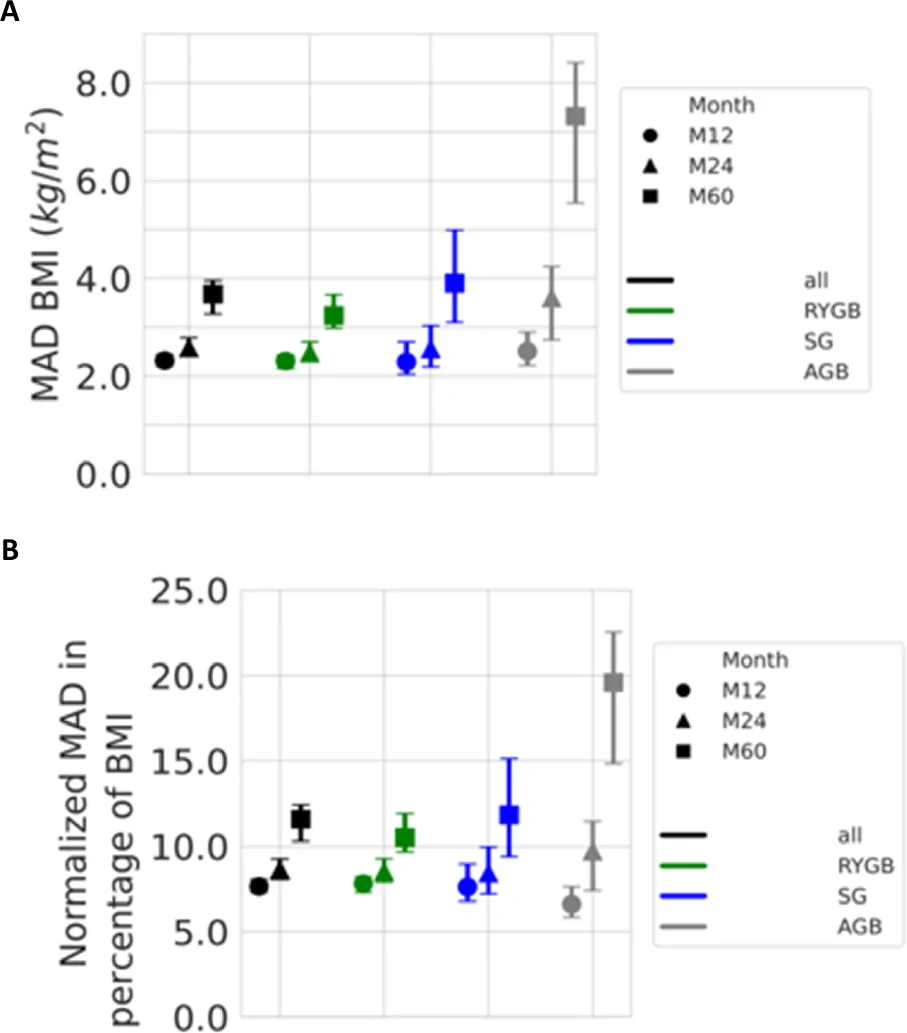
Accuracy of body mass index prediction according to the surgical technique at 12, 24, and 60 postoperative months. The accuracy of the prediction is estimated by calculating the difference between predicted and observed BMI for a typical patient, and expressed as the median absolute deviation (MAD). To facilitate clinical interpretation, **A** illustrates the results as absolute values of body mass index (kg/m^2^), and the **B** summarizes the results as the percentage of the predicted body mass index (%). The lower the MAD, the more accurate the prediction. BMI body mass index, AGB adjustable gastric band, RYGB Roux-en-y gastric bypass, SG sleeve gastrectomy.

When we analyzed the respective role of the seven predictive features previously selected in adults [19], using decision trees, the surgical technique was the only parameter significantly associated with 5-year weight loss prediction. For earlier visits (up to 2 years), the presence of diabetes impacted weight loss negatively for patients who underwent SG and RYGB. There was no other meaningful predictive feature for weight loss after AGB at any postoperative time. Smoking status had no influence on weight loss trajectories. The model’s prediction performances were evaluated at 1, 2, and 5 years in the testing subset of the training cohort. Overall, the mean difference between predicted and observed BMI at 5 years was 3.7 kg/m² (95% CI 3.3–3.9), resulting in a 12% normalized difference. The predictive performances of the model at 1 year, 2 years, and 5 years in the testing cohort and for each intervention are shown in Fig. 3. At 5 postoperative years, predictive performances were significantly better after RYGB (MAD 3.2) than after AGB (MAD 7.3, *p* < 0.001), and moderate after sleeve gastrectomy (MAD 3.9, *p* = 0.06) (Fig. 3).

**Fig. 4:**
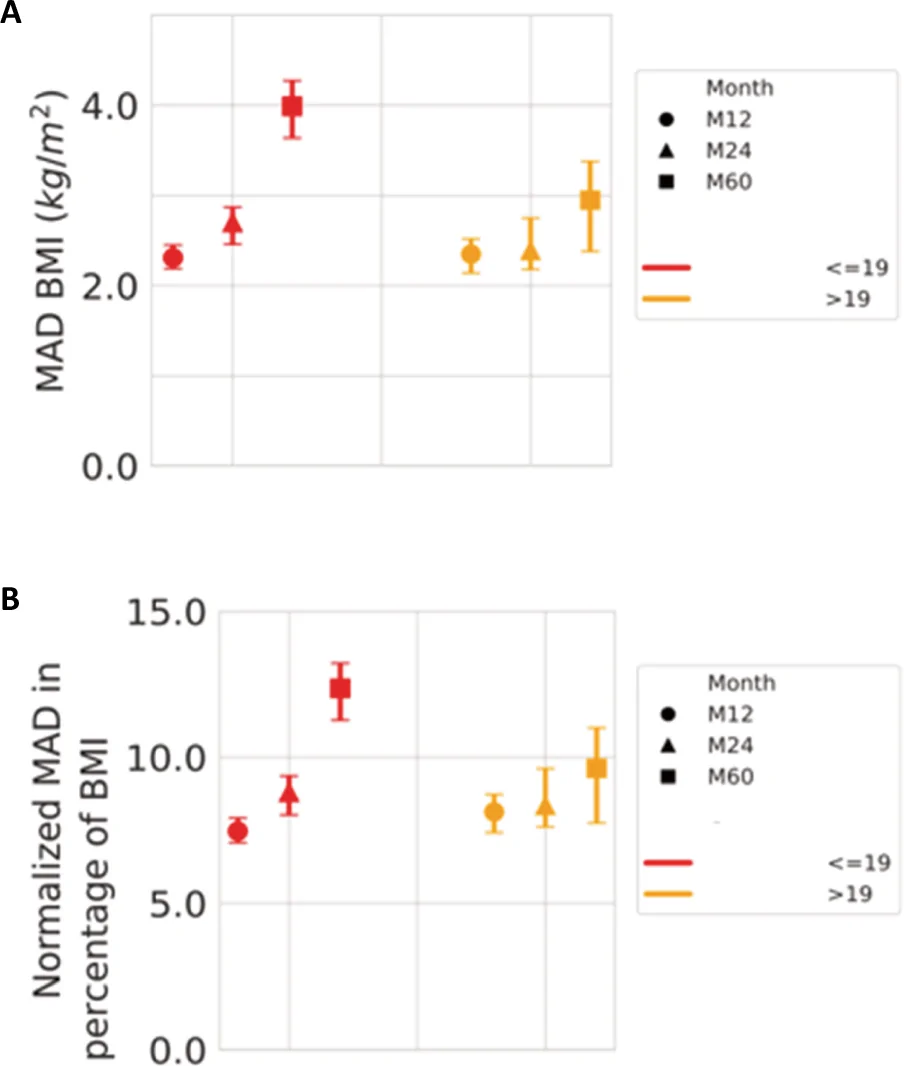
Body mass index prediction accuracy according to age: adolescents (age ≤ 19 years) compared to young adults (age > 19 years) at 12, 24 and 60 postoperative months. The differences between predicted and observed BMI for each subgroup is expressed as the median absolute deviation (MAD) of body mass index (**A**) and normalized MAD in percentage of the predicted body mass index (**B**). Younger age was associated with less accurate prediction. BMI body mass index.

Finally, we evaluated how the age at the time of surgery influences the accuracy of weight loss prediction. We defined as a cut-off the median age of the cohort (19 years). As presented in Fig. 4, the accuracy of weight loss prediction was lower in younger participants (higher MAD under 19 years).

Finally, the machine learning model developed in the present study for adolescent was integrated into the existing software (https://bariatric-weight-trajectory-prediction.univ-lille.fr/). The resulting user-friendly calculator displays the predicted weight trajectory at any given time during 5 years after surgery, alongside prediction intervals corresponding to IQR of prediction errors. By default, individual predicted trajectories are expressed in kilograms. According to the user’s choice, results can also be displayed in kg.m^-^², percentage of TWL, or percentage of Excess Weight Loss. To improve readability, predicted trajectories over time for each of these metrics are displayed as smooth curves.

**Fig. 5:**
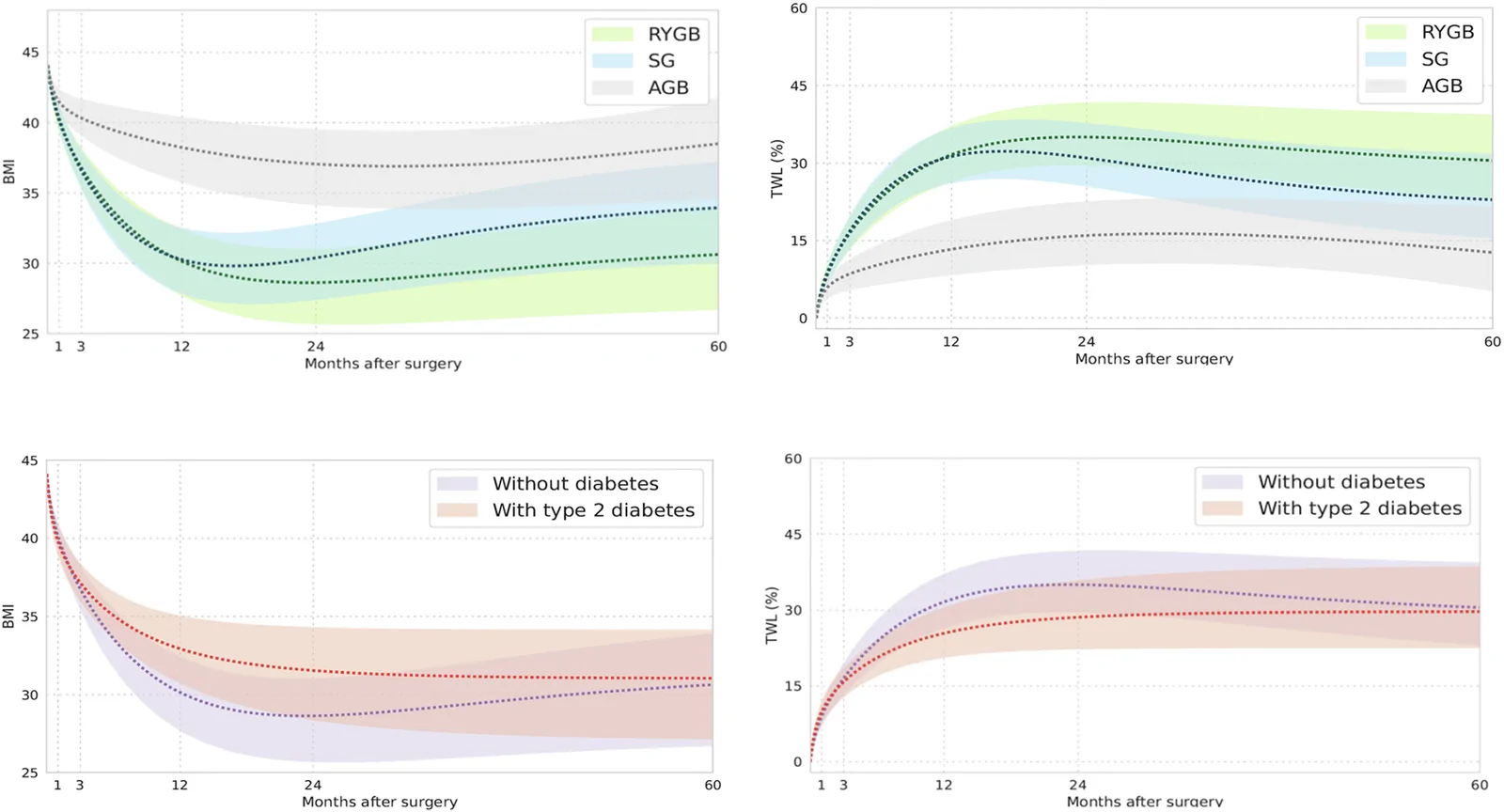
Examples of predicted weight loss trajectories for a 5-year period. Upper panels illustrate the prediction of weight loss trajectories according to different surgical techniques and obtained for a 16-year-old patient, without diabetes, weighing 120 kg for a height of 165 cm. The upper left panel shows BMI trajectories and upper right panel shows total weight loss trajectories. Lower panels illustrate the prediction of weight loss trajectories after RYGB, obtained for a 16-year-old patient, with or without diabetes, weighing 120 kg for a height of 165 cm. The lower left panel shows BMI trajectories and lower right panel shows total weight loss trajectories. AGB adjustable gastric band, BMI body mass index, RYGB Roux-en-y gastric bypass, SG sleeve gastrectomy, TWL total weight loss.

Two illustrative examples of BMI and TWL trajectories predicted for individual adolescent patients from the updated version of the bariatric weigh trajectory prediction calculator are shown in Fig. 5.

## Discussion

In this study, we adapted and validated in adolescents a machine learning-based model, previously developed in adults to predict weight loss trajectory after bariatric surgery. The resulting calculator was able to predict 5-year BMI in adolescent with a 12% precision, as compared to the observed values, based on collection of five simple preoperative variables, namely age, weight, height, type of intervention, and type 2 diabetes status.

The updated model, based on one of the largest reported cohorts of adolescents undergoing bariatric surgery [24–26], is the first validated predictive tool specifically adapted for this population. While some authors have identified factors beyond baseline BMI that may influence the magnitude and durability of postoperative weight loss—such as the duration of preoperative preparation or intentional weight loss before surgery [8, 27, 28]—these findings have not been consistently replicated elsewhere [29]. Research on predictors of postoperative weight loss in adolescents remains limited, and existing studies provide only modest evidence due to relatively small cohort sizes.

Compared to adults, the adapted calculator showed a lower, but still clinically relevant accuracy for predicting 5-year BMI in youth (MAD 3.7 kg m^−2^ vs. 2.8 kg m^−2^ in adults) [19]. Surgical technique had a major influence on the accuracy of the weight loss prediction, which was maximal for RYGB, moderate for SG, but much lower for patients who had an AGB. Differences in weight loss between surgical techniques have been already reported by others in adolescents, and the choice of the optimal surgical approach remains debated. In the present study, we found that long-term weight loss was markedly superior and more predictable following RYGB than after AGB [15, 24, 25, 30–41].

Of note, the accuracy of prediction was also lower in younger patients (patients under 19). It also decreased with the duration of follow-up, as already highlighted by Inge et al. [42]. In adult cohorts, younger age has been described as a favorable factor for weight loss [43], although weight regain is more common in younger patients, resulting in a greater dispersion of results with time [44]. The explanation of this finding remains elusive [26]. It is important to consider that adolescents undergo unique physiological and psychosocial development, which might not be fully captured by the features derived from the adult model. We cannot exclude that the proposed model could be missing significant adolescent-specific predictors.

Additionally, the presence of type 2 diabetes negatively affected weight loss during the initial postoperative years. Type 2 diabetes was, however, rare (3.4%) in adolescents and its duration at the time of surgery was shorter as compared to adults. Our results are consistent with findings of Borges-Canha et al., who reported that insulin resistance and beta cell dysfunction impact negatively post-surgical weight loss, even before type 2 diabetes onset [45]. In adults, type 2 diabetes is known to be a major limiting factor of weight loss after bariatric surgery, particularly when the disease is long-standing and poorly controlled [19, 46, 47]. Interestingly, postoperative weight loss trajectories converged at 5 years between patients with and without diabetes at baseline (Fig. 5). This result illustrates that in adolescents, the negative impact of type 2 diabetes on weight loss disappeared with time, in line with other reports showing sustainable diabetes resolution after bariatric surgery in that age group [15, 25, 37, 42].

Our study had several limitations. First is its retrospective design, and the lack of comprehensive clinical, biological, and genetic parameters analyzed. Furthermore, we did not analyze social, developmental, or psychological aspects which may differ significantly between adolescents with severe obesity and their normal-weight peers [48]. Third, all study participants were enrolled in two European countries, and a vast majority were Caucasian. In contrast to its initial adult version [19, 49], the present updated calculator was not externally validated in adolescents with other genetic backgrounds which may have a negative impact on long-term weight trajectories [50–53]. Therefore, the accuracy of the proposed tool remains to be tested on additional independent cohorts of adolescents with greater ethnic diversity [9].

In summary, we adapted a machine-learning–based tool originally developed for adults and demonstrated that the updated version accurately predicts weight loss after bariatric surgery in adolescents. Our findings indicate that surgical technique—and, to a lesser extent, age and type 2 diabetes—influence postoperative weight-loss trajectories as well as the accuracy of their prediction in adolescents and young adults. Finally, the updated calculator can be integrated into routine clinical practice to support shared decision-making and enhance discussions between healthcare providers and young patients considering bariatric surgery.

## Data Availability

The two datasets generated or analyzed during the current study are not publicly available because they are subject to national data protection laws and restrictions imposed by the ethics committee to ensure data privacy of the study participants. However, access to the datasets can be applied for through an individual project agreement with principal investigators of the French cohort (iva.gueorguieva@chu-lille.fr or francois.pattou@univ-lille.fr) or the Swedish cohort (torsten.olbers@liu.se).
